# Integrative analysis of hi-C variance and multi-contact data identifies enhancer–promoter hub structures

**DOI:** 10.1093/bib/bbaf631.039

**Published:** 2025-12-12

**Authors:** Hideki Tanizawa, Claire Yik-Lok Chung, Ken-ichi Noma

**Affiliations:** Institute for Genetic Medicine, Division of Genome Biology, Hokkaido University; Institute for Genetic Medicine, Division of Genome Biology, Hokkaido University; Institute for Genetic Medicine, Division of Genome Biology, Hokkaido University; Department of Biology, Institute of Molecular Biology, University of Oregon

## Abstract

Enhancer–promoter (EP) interactions are essential for transcriptional regulation, yet the mechanisms underlying the simultaneous engagement of multiple enhancers with a single promoter (‘hub structures’) remain elusive. Here, we present a computational framework that integrates Hi-C variance analysis with multi-contact data to identify such structures. We statistically modeled replicate-to-replicate variability in Hi-C contact frequencies, enabling the extraction of dynamically flexible genomic regions that are not evident from averaged contact maps. This variance-based approach highlights loci with high interaction plasticity, which often coincide with regulatory elements. To further characterize these regions, we incorporated multi-contact information from Pore-C and active EP association signals from H3K27ac-HiChIP, allowing us to detect EP hubs and evaluate their molecular determinants. Our comparative analysis revealed distinct mechanisms: Condensin tends to recruit multiple enhancers to a promoter simultaneously via a diffusion capture–based process, whereas Cohesin predominantly mediates one-to-one interactions through loop extrusion. This difference suggests that Condensin contributes to cooperative enhancer activity, particularly in contexts where strong transcriptional upregulation is required. In this presentation, we will introduce the framework, demonstrate its application to yeast and human fibroblast datasets, and discuss its potential for dissecting dynamic features of genome architecture at scale.

